# The VRNetzer platform enables interactive network analysis in Virtual Reality

**DOI:** 10.1038/s41467-021-22570-w

**Published:** 2021-04-23

**Authors:** Sebastian Pirch, Felix Müller, Eugenia Iofinova, Julia Pazmandi, Christiane V. R. Hütter, Martin Chiettini, Celine Sin, Kaan Boztug, Iana Podkosova, Hannes Kaufmann, Jörg Menche

**Affiliations:** 1 CeMM Research Center for Molecular Medicine of the Austrian Academy of Sciences, Vienna, Austria; 2 Department of Structural and Computational Biology, Max Perutz Labs, University of Vienna, Vienna, Austria; 3 Ludwig Boltzmann Institute for Rare and Undiagnosed Diseases, Vienna, Austria; 4 St. Anna Children’s Cancer Research Institute (CCRI), Vienna, Austria; 5 St. Anna Children’s Hospital, Department of Pediatrics and Adolescent Medicine, Medical University of Vienna, Vienna, Austria; 6 Department of Pediatrics and Adolescent Medicine, Medical University of Vienna, Vienna, Austria; 7 Institute of Visual Computing and Human-Centered Technology, TU Wien, Vienna, Austria; 8 Faculty of Mathematics, University of Vienna, Vienna, Austria

**Keywords:** Computational platforms and environments, Network topology, Software, Molecular medicine, Biomedical engineering

## Abstract

Networks provide a powerful representation of interacting components within complex systems, making them ideal for visually and analytically exploring big data. However, the size and complexity of many networks render static visualizations on typically-sized paper or screens impractical, resulting in proverbial ‘hairballs’. Here, we introduce a Virtual Reality (VR) platform that overcomes these limitations by facilitating the thorough visual, and interactive, exploration of large networks. Our platform allows maximal customization and extendibility, through the import of custom code for data analysis, integration of external databases, and design of arbitrary user interface elements, among other features. As a proof of concept, we show how our platform can be used to interactively explore genome-scale molecular networks to identify genes associated with rare diseases and understand how they might contribute to disease development. Our platform represents a general purpose, VR-based data exploration platform for large and diverse data types by providing an interface that facilitates the interaction between human intuition and state-of-the-art analysis methods.

## Abstract (web summary)

Data-rich networks can be difficult to interpret beyond a certain size. Here, the authors introduce a platform that uses virtual reality to allow the visual exploration of large networks, while interfacing with data repositories and other analytical methods to improve the interpretation of big data.

## Introduction

**Fig. 1:**
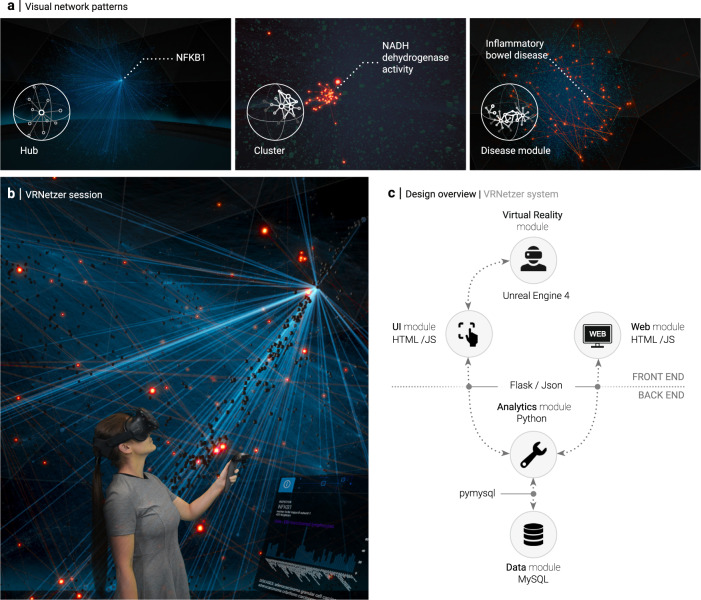
A Virtual Reality (VR) network exploration platform. **a** Three examples of visual network patterns with direct biological interpretations. Left: highly connected hub, indicating essential and often pleiotropic gene functions. The depicted *NFKB1* gene is a subunit of an essential transcription factor involved in a wide variety of biological processes. Middle: small, densely interconnected clusters often represent a particular biological function. The highlighted cluster corresponds to the NADH-dehydrogenase, the first complex of the electron transport chain. Right: genes associated with the same disease are often characterized by larger, yet significantly connected modules, as shown for genes associated with inflammatory bowel disease. **b** Green-screen composition of a user wearing a VR headset and the respective VR scene she is currently exploring. **c** The VRNetzer platform consists of five modules that can be customized independently. The frontend consists of the VR interface and an additional web browser-based interface to facilitate seamless exchange between VR sessions and standard working environments. The user interface module connects the VR module to the backend, which consists of two separate modules for data storage and data analytics. The individual modules are implemented in programming languages that are widely used for the respective tasks.

Network theory provides an arsenal of powerful tools and concepts for analyzing diverse data. In biology and medicine, it has found numerous applications for disentangling the enormous complexity within and across different levels of biological organization^1–4^. Networks have a distinct advantage compared to other computational methodologies for integrating and interpreting biological data: Their visual representation allows for a uniquely intuitive interpretation, enabling us to quickly identify potential local and global patterns in complex data that can then be further assessed by advanced computational and statistical means (Fig. 1a): In molecular interaction networks, for example, highly connected hubs generally correspond to genes that play important roles in healthy and disease states, such as pleiotropic genes^5^ or cancer driver genes^6^. Dense local agglomerations of nodes often correspond to specific biological functions^7,8^, and also disease-associated processes are characterized by distinct connection patterns^1,9^. Interactive visualizations of such interaction patterns are thus a core component of major web resources and databases for exploring functional annotations of individual genes or small gene sets^10–14^. Also static visualizations of large-scale biological networks may yield important insights into the overall architecture of the represented system, leveraged for example in large-scale mapping efforts of biological interactions^5,7,8,15,16^.

Numerous dedicated softwares are available for small interactive, or large static network visualization^17–23^^.^ These softwares may also include a wide range of additional features, such as the possibility to implement custom analysis methods or additional data sources (see “Methods”). However, the full potential of networks is still constrained by several fundamental challenges: Large networks exhibit functionally relevant structures at various scales, ranging from small node clusters (representing, e.g., protein complexes) to mesoscale structures (e.g., disease associated neighborhoods) and global patterns (e.g., node centralities). The inherent multi-scale nature of biological processes can only be fully appreciated when the entire range from local to global network structures can be inspected continuously and interactively. Until now, this is only possible for very limited network sizes of up to a few hundred nodes. On a conventional computer screen, networks beyond this size typically lead to unintelligible hairball visualizations that obscure existing structure and offer little insights. This requires the selection of a smaller subnetwork, yet in many applications, it is not clear how to select the most meaningful one. For example, the network neighborhood around a particular gene that describes the pathobiological mechanisms of a disease in which it is involved is generally different from the neighborhood of its normal, homeostatic function^9^. The small-world property of biological networks implies that large parts of the entire network can be reached within a few steps only, making selections of small subnetworks a difficult process that may discard an important intrinsic property of the architecture of biological systems.

Here we describe an approach to address these challenges by using immersive Virtual Reality technology (Fig. 1b). The immersive nature of VR provides a sense of depth that cannot be achieved by other means, such as 2D or non-immersive 3D representations. This allows for quickly and accurately resolving connections that would otherwise be ambiguous due to partially occluded nodes and/or links. As a result, network size orders of magnitude larger can be meaningfully visualized, thus enabling the smooth and continuous inspection of both local and global network structure. We present network layouts specifically tailored to leverage this immersive environment and allow for interactive exploration of different functional and structural network characteristics. As a proof of concept, we show how this exploration can be leveraged to investigate gene variants in the context of a molecular interaction network for identifying variants responsible for severe genetic diseases.

## Results

### Design overview of the VRNetzer platform

Our VRNetzer platform is designed in a modular fashion, allowing the user to customize and extend the components for data visualization, data analysis, and data input separately. The platform consists of five key modules illustrated in Fig. 1c: (1) All data are stored and organized in a single data module implemented as a standard MySQL database. We included a molecular network curated from the literature, as well as widely used gene annotation data ranging from molecular functions to disease associations. (2) The analytics module is used to access the data, perform user-defined analyses via a Python interface and communicate the results to the interactive data exploration frontends. (3) The core of our VRNetzer platform is the VR module, providing an immersive 3D frontend for visualizing and exploring large networks. It is implemented in the industry-standard Unreal game engine^24^, providing highly performant graphics rendering on non-specialized and relatively inexpensive computer hardware, as well as maximal compatibility with a broad range of VR hardware. The VR module is connected to the data analytics engine via a (4) user interface (UI) module that serves as a communication layer and offers the functionality of implementing arbitrary UI elements using standard web design libraries. We also provide a (5) web module as a browser-based frontend designed for tasks that can be performed more conveniently on a conventional computer screen, such as data preprocessing or further inspection of the results of a VR exploration session. The web module thus facilitates the integration of VR exploration into established workflows and can also be used as a standalone application when no VR hardware is available.

### Immersive network exploration

**Fig. 2:**
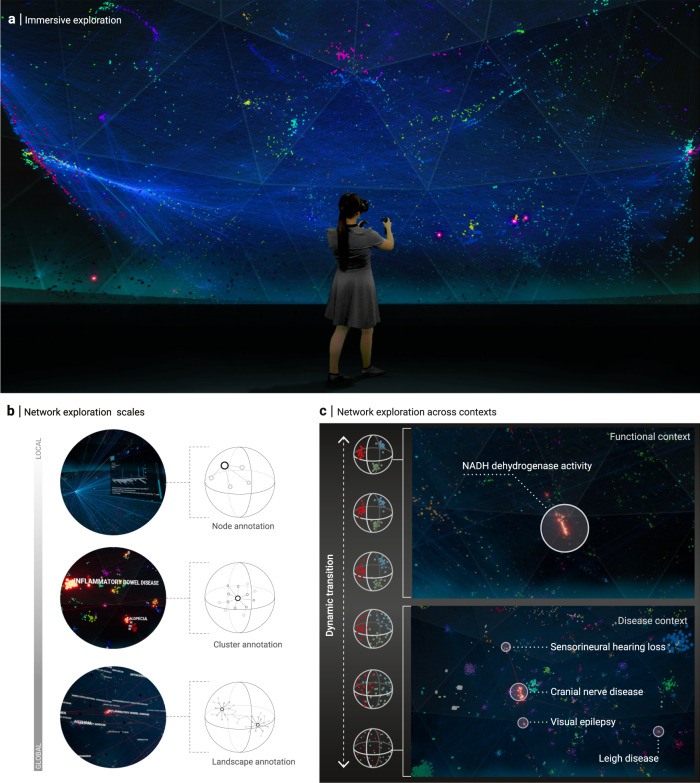
Immersive network exploration. **a** Green-screen composition showing a user immersed in the genome-scale molecular interaction network. **b** Network annotations at different scales: individual nodes are annotated with detailed information shown in a separate inspection panel. Meso-scale node clusters (representing, for example, biological functions or disease-associated processes) are annotated using floating labels derived from gene set enrichment and network proximity. Functional landscapes represent global network views in which neighborhoods and their respective annotations are highlighted. Our platform includes functional landscapes for gene annotations according to gene ontology (GO) and disease associations. **c** The dynamic transition functionality allows for exploring different roles of a particular gene or gene set across different contexts. Starting, for example, in a functional landscape showing the biological processes that a gene set is involved in (here: NADH-dehydrogenase activity; top panel), the positions of all nodes smoothly transition toward a functional landscape that highlights disease neighborhoods in which the respective genes are located.

Conventional representations on a computer screen limit the size of networks that can be explored in an interactive fashion to a few hundred nodes and links. Our immersive VR platform, in contrast, allows for seamless navigation within genome-scale molecular networks, such as the human interactome consisting of around 16,000 proteins as nodes and around 300,000 physical interactions as links (Fig. 2a). The most basic navigation modes are the free movement within the surrounding network, rotation, and translation of the network with full six degrees of freedom, and arbitrary scaling of the network size. The scaling allows for a seamless transition between global network views and close-up inspection of local node clusters or individual nodes.

Our platform provides functionality to annotate and analyze the network for each level of organization: Individual nodes can be inspected via a panel displaying any information contained in the underlying database and by highlighting all directly connected neighbors (Fig. 2b, top). Node clusters can be annotated with attributes from the database, such as enriched gene ontology (GO) terms or disease associations (Fig. 2b, middle). To realize the full potential of our platform for global visualizations of large networks, we developed a series of network layouts that are specifically designed for immersive exploration. These layouts allow us to assign explicit meaning to absolute and relative node positions in 3D space, both on the level of node annotations and the level of structural network characteristics: In functional landscape layouts, the nodes are clustered together based on a combination of network proximity and annotation similarity, representing, for example, network neighborhoods associated with a particular disease (Fig. 2b, bottom). Compared to conventional network layouts, such as those generated by force-based algorithms, in which the positioning of nodes is often only indirectly linked to structural or functional network characteristics, our layouts offer an explicit and thus easily interpretable spatial organization. By observing the dynamic transition between different explicit layouts, i.e., by gradually moving all nodes from their position in one particular layout to new positions in another, we can investigate nodes and their properties in different contexts (Fig. 2c). For example, we can follow a selected group of nodes from a layout emphasizing their biological function (Fig. 2c, top) to a layout showing their involvement in different diseases, while simultaneously inspecting their structural network properties, such as their degree (Fig. 2c, bottom).

Taken together, these immersive exploration features allow users to quickly locate individual nodes and node clusters, identify their immediate and broader neighborhoods in different functional contexts, while seamlessly moving between network scales, from individual nodes to the whole network.

### User interaction design

**Fig. 3:**
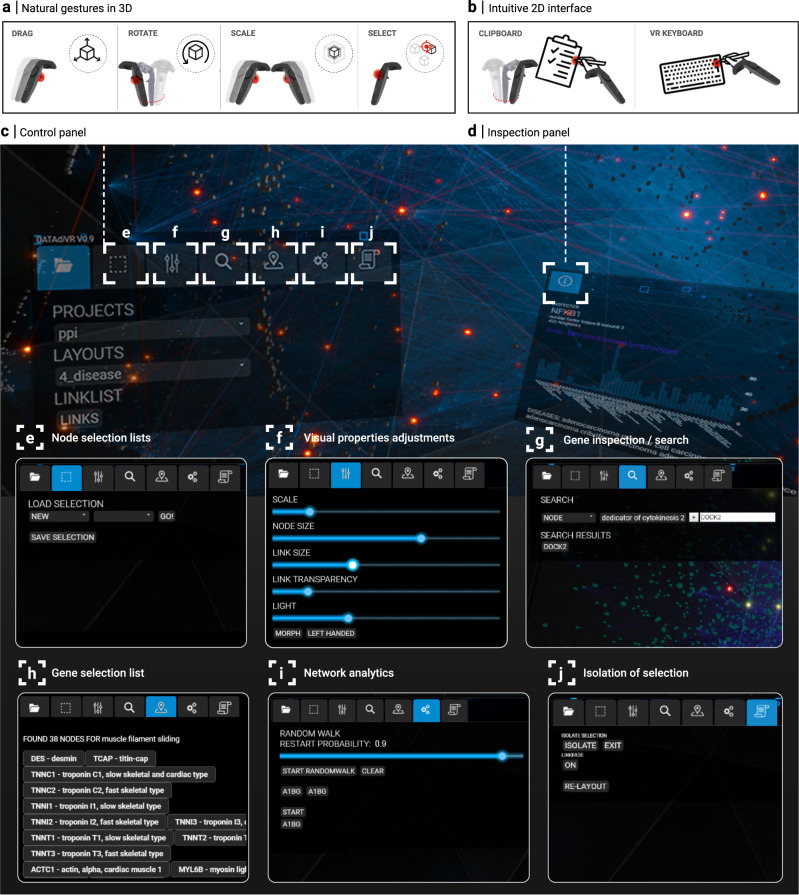
User interaction design. **a** Interactions with the 3D network, such as moving the network or selecting individual nodes, are implemented as natural 3D gestures. The movement of the controllers with pressed trigger buttons (indicated in red) is directly translated into the corresponding movement of the network, allowing the user to drag, rotate, and scale the network. Individual nodes are selected by directly pointing at them. **b**, All other interactions are performed through 2D interfaces dedicated to specific tasks. The core functionalities are collected into a control panel that spawns as a virtual clipboard in one hand, and a virtual stylus in the other, which can also be used to type on a virtual keyboard. VR screenshots showing the (**c**) control panel and a particular (**d**) inspection panel. Inspection panels are used to display the results of a particular action or analysis, such as providing detailed annotations for a selected node. **e**–**j** Screenshots of the different core functionalities, organized as different tabs in the control panel. **e** Loading (saving) specific node sets from (to) the database. **f** Adjusting visual properties of the network. **g** Searching for nodes and node sets, either by name or any other attribute contained in the database. Searches can also be combined using logical operators. **h** List of currently selected nodes. **i** User interface for running network analytics, such as random walk with restart from a selected node set. **j** Highlighting node selections.

Our goal was to create a UI that is as self-explanatory as possible by utilizing familiar concepts from 2D interfaces, while also making use of the unique, yet unfamiliar capabilities of VR. We use standard HTC Vive controllers as interface devices, providing six degrees of freedom regarding their absolute position and rotation in space, together with several clickable buttons. The controllers allow for manipulating 3D objects through natural gestures, for example moving and rotating the network or selecting individual nodes (Fig. 3a). For more programmatic tasks, such as selecting from a list of analytical methods, we designed a virtual clipboard for displaying 2D panel interfaces, together with a virtual stylus for interacting with them (Fig. 3b). For the panel interface design, we could build on 2D UIs familiar from computer screens.

We introduce two main panels, each equipped with different tabs: A control panel and an inspection panel (Fig. 3c, d). The control panel is the primary interface for network exploration tasks, including loading networks and node selections (Fig. 3c, e), adjusting visual properties (Fig. 3f), searching for nodes or node sets matching certain attributes contained in the database (Fig. 3g, h), performing network analysis tasks (Fig. 3i), and working with subnetworks (Fig. 3j). The inspection panel is used to display more detailed information on specific tasks and results of network analyses.

By the modular design of our platform, the UI not only serves as an interface between the user and the platform, but also as a communication layer between the VR and web frontend modules and the analytics backend module: For example, when a button is pressed in the VR, a request is sent to the analytics module, whose response, in turn, is passed to the VR module for rendering. We implemented the UI as a simple JavaScript webpage, allowing users to easily extend the existing functionality without any knowledge of the VR engine. Any UI element, such as dashboards, buttons triggering specific actions in the analytics module, as well as additional data visualization elements, such as graphs or bar charts, can be implemented using standard libraries, such as D3.js.

### Integrating immersive exploration in standard workflows

Our platform is also equipped with a web-based frontend that does not require any VR hardware and can be accessed through a standard web browser. The web module uses the same design as the control and inspection panels inside the VR environment and accesses the same database and analytical functionalities, for example, to create and review selections, perform searches, explore attributes, and run network analyses. It, therefore, offers a convenient interface to perform tasks for which the VR environment is impractical, or when no VR hardware is available. It further enables easy integration of VR-based modes of exploration with established workflows, computational tools and resources. In particular, the web module serves as an interface to load new data into the platform. The import tool includes a data validation function to ensure the consistency of the uploaded data. In preparation for a VR session, new networks can be uploaded, and existing ones updated with additional nodes, links, or attributes, such as associated diseases or patient variants. Similarly, the web module can be used after a VR session to download, review and annotate the results, and to share them with collaborators.

### Data infrastructure

The core data infrastructure consists of a MySQL database and a Python Flask-based web server, which may run on the same machines as the VR and web modules, or on separate ones. This allows for easy integration of existing data in diverse formats using standard tools, as well as convenient post-processing of results from VR sessions. We provide a range of network and node annotation data. As a molecular network dataset we include the human interactome network of protein–protein interactions as curated from the HIPPIE database^25^. It compiles all known physical protein interactions, resulting in a largest connected component of 16,376 proteins linked by 309,355 connections. In addition, we have pre-populated the database with various frequently used gene annotations. Specifically, we have included known associations between genes and diseases^26–28^ or phenotypes^29–31^; between genes and biological processes, molecular functions, and cellular components^32,33^; pathway annotations^11–13^; data on tissue-specific gene expression^34^; and abstracts of articles from PubMed relevant to specific genes^35^.

### Implementation of data analysis methods

The analytics module serves as an intermediary between front- and backend modules and allows for implementing custom data analysis methods. It is written in Python, thus enabling the easy integration of numerous powerful data analysis packages into our platform. We implemented a range of functionalities, representing both general network exploration tasks, as well as tasks specific to the application of candidate gene prioritization presented below: Genes and gene sets can be retrieved based on attributes contained in the database, such as functional or disease annotations. For more complex searches, up to four AND and OR clauses can be combined. Since biological annotations are often organized in hierarchical taxonomies, for example, the GO, we also included these relationships into our search functionality. Likewise, annotations of selected genes can be retrieved, either directly or using gene set enrichment analysis. Network-based functionalities include the identification of a shortest path between two nodes, identification of connected subgraphs among selected nodes, re-layout of selected nodes, and neighborhood expansion via random walk with restart^36^.

### Application to prioritization of genomic variants

**Fig. 4:**
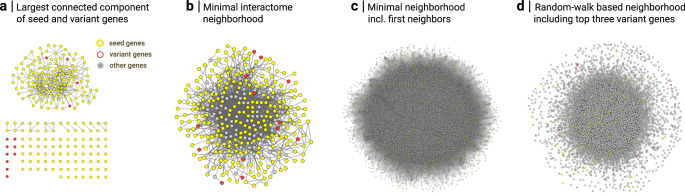
Static 2D visualization of the seed and variant genes in the interactome. **a** The 247 seed and 13 variant genes are scattered across several disconnected subnetworks in the interactome. The largest connected component contains 157 genes (153 seed and 4 variant genes). **b** Using a Steiner tree approach^60^, we can identify a minimal set of linker genes for integrating all seed and variant genes into a single connected component. The resulting subnetwork consists of *N* = 334 nodes and *M* = 1387 links. **c** Expansion of the subnetwork in (**b**) with all first neighbors results in *N* = 10,915 nodes and *M* = 261,853 links. **d** Random walk-based interactome neighborhood around the seed genes obtained by including all genes that are ranked above the top three variant genes (*N* = 4052; *M* = 118,218). All network visualizations were created using the Cytoscape software.

**Fig. 5:**
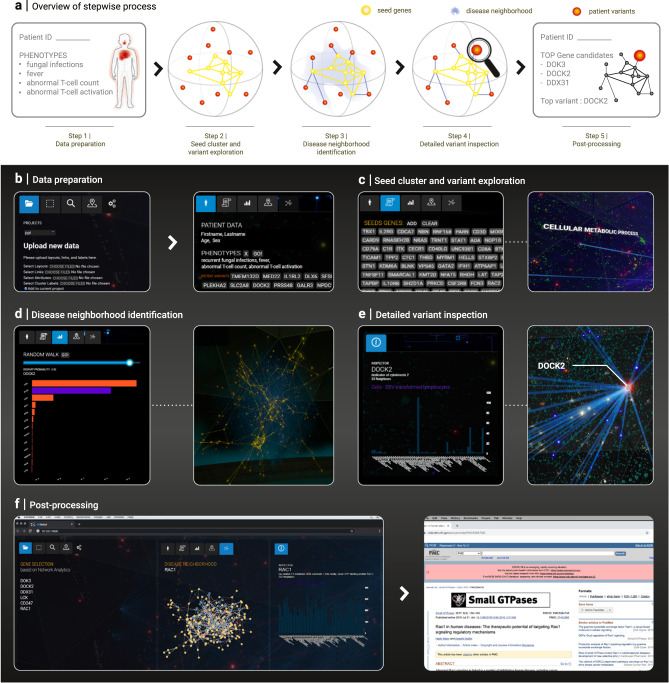
Using the platform for prioritizing genomic variants of a rare disease patient. **a** Overview of the different steps of the procedure, from collecting relevant data in the web module to performing interactive network analyses in VR and finally post-processing the results. **b**–**f** Screenshots illustrating the individual prioritization steps. **b** The required data (patient phenotypes and candidate genes, seed genes, network data) are collected and curated using the web interface. All data are immediately available also in the VR environment thanks to the shared data module infrastructure. **c** Individual candidate genes (red) and seed genes are inspected in their network context to identify related biological functions and known disease associations. For example, the blue links show a connection between the candidate gene *DOCK2* and cellular metabolic processes in the functional landscape corresponding to GO biological processes. **d** The broader network neighborhood around the seed genes is identified using a random walk with restart algorithm, providing a provisional and purely network-based ranking of the candidate genes. Left: inspection panel showing a bar plot of the resulting visiting frequencies of the 13 candidate genes. Right: the seed genes (yellow) are significantly connected, indicating a particular network neighborhood associated with the respective disease. **e** Detailed inspection of the most highly ranked candidate genes according to known functions and disease associations within their network neighborhood. Left: Inspection panel showing expression levels of *DOCK2* across different tissues. Right: Connections of *DOCK2* in the network view. **f**, Saving the key results of the VR exploration for further inspection and follow-up analyses in the web module. Left: Screenshot of the web module summary of the VR session. Right: Following up on a particular interaction partner of DOCK2, RAC1, in PubMed.

Molecular networks are an indispensable tool for addressing a wide range of biomedical research questions^4^. Examples range from understanding the essentiality of individual genes^37,38^ or the biological function of interacting proteins^7^ to elucidating the molecular mechanisms of complex diseases^3^ or disease comorbidities^1,39^. A particularly successful translational application of molecular networks is aiding in the genetic diagnosis of patients suffering from rare diseases that are caused by a single gene defect. The starting point is next-generation sequencing of a patient’s genome, typically resulting in hundreds of candidate genes containing variants of unknown significance. Scoring and prioritizing these candidate genes to single out the one causal gene defect represents a major bottleneck in the genetic diagnosis process. A number of methods have been developed for prioritizing genes based on their context in molecular networks, often augmented by additional clinical data^40–45^ (see “Methods” for a discussion of existing tools). These methods rank candidate genes according to the network proximity of their products to a given set of seed genes, i.e., genes known to be implicated in a certain phenotype or disease. This is based on the fundamental observation that proteins associated with the same disease are not scattered randomly in molecular networks but aggregate in disease-specific neighborhoods or ‘disease modules’^1,46^. While network-based ranking methods now represent the state-of-the-art in disease gene prioritization, further inspection of the resulting putative list of top candidates is still required. Geneticists and clinical experts of the particular disease phenotype will evaluate each top candidate individually, e.g., regarding known gene functions, interaction partners or expression patterns in relevant cell types or tissues, to identify the most promising gene and putative molecular mechanism of the disease. This process is often time consuming and performed in an iterative fashion involving both computational and disease experts, such that an initial exploration of a candidate list may suggest a refinement in the parameters of the prioritization method. A visual inspection of the respective network neighborhoods could facilitate this process, but is hampered by the large network sizes that are typically involved at this stage, since the biological context of every candidate gene (i.e., the respective network neighborhood of its product) needs to be carefully evaluated in order to identify the most promising candidates. Due to the highly connected nature of the interactome, even a relatively small number of seed and variant genes can quickly generate a very large number of connected gene products (Fig. 4). Filtering for more manageable subnetworks a priori is difficult not only because all connected proteins are potentially relevant, but also because different subnetwork identification methodologies may generate very different outcomes. Our platform enables inspecting the full network context and is therefore ideally suited for an interactive exploration performed by a disease expert, even without specialized computational knowledge. We propose a five-step procedure outlined in Fig. 5a and apply it to previously published data^47^ from a specific patient.

Step 1: Data preparation—we start by collecting and curating all required data, specifically phenotypic and genomic patient information, a set of related seed genes, and molecular interaction data. This step is most conveniently performed in the browser-based web module (Fig. 5b), allowing for a seamless integration into a user’s standard workflows.

We consider a patient suffering from severe combined immune deficiency with an unknown genetic cause^47^. The patient was first hospitalized by the age of 3 months, presenting with recurrent severe infections, fever, and oral moniliasis. An immune panel further revealed a reduced number of T cells and defective T-cell activation. The patient underwent next-generation whole-genome sequencing. The resulting genomic variants were filtered according to quality and frequency, and scored for deleteriousness using standard tools as detailed in^47^. The final list of variants, validated by Sanger sequencing and segregation analysis, corresponded to 15 candidate genes.

Seed genes are typically compiled from genes associated with similar diseases or phenotypes. Our platform provides associations from HPO (human phenotype ontology)^29–31^, DisGeNET^26^ and OMIM (Online Mendelian Inheritance in Man)^28^ that can be searched and further inspected to compile a set of seed genes that best fits the particular use case, for example using the HPO terms corresponding to the observed clinical phenotypes. In addition, also custom seed gene sets curated outside of the platform can be uploaded. For the specific patient considered here, we use an expert-curated list of 276 genes that underlie inborn errors of immunity taken from^48^ as seed genes.

Finally, we use the HIPPIE database^25^ to compile an interactome network consisting of 16,376 gene products and 309,355 interactions. The interactome contains 247 out of 276 seed genes and 13 out of 15 candidate genes.

Step 2: Initial exploration of patient data and disease context in VR— Since both frontends share the same data infrastructure, all network data and gene lists curated in the web module are immediately available within the VR module. For a first orientation within the molecular network, we inspect individual candidate genes and seed genes. This can be done by selecting genes of interest in the respective UIs (Fig. 5b, c), by searching for the name of a particular gene (Fig. 3g) or by directly clicking on a node in the network. The inspection panel summarizes the information available in the database, such as known functional and disease annotations, expression levels in different tissues, as well as connectivity information. We can explore the broader network context of a particular gene by switching between different functional layouts using the dynamic transition functionality and inspecting the annotated clusters in which the gene lies (Fig. 2c). For example, an inspection of the position of different candidate gene products within the GO cellular component centric functional layout reveals quickly that DOK3 is part of a cluster annotated with the cytoplasmic side of the plasma membrane, that DOCK2 is localized in the cytoplasm, and DDX31 in secretory granules, respectively. Note that also connected neighbors of the selected proteins can be easily followed throughout the dynamic layout transitions via the highlighted links (Fig. 5c). This increases the amount of contextual information that can be quickly assessed substantially compared to gene-level inspection of conventional annotation tools. For example, visual inspection of variants and their connections in different functional layouts suggests that several variants (DOCK2 and DOK3) have connections with network neighborhoods associated with cellular metabolism (Fig. 5c), activation of immune response (DOCK2) or ER stress (DOK3).

Step 3: Disease neighborhood identification—Network-based approaches for prioritizing candidate genes rely on the existence of a well-localized seed cluster. We can use the selection isolation and highlighting features to visually confirm that the products of the chosen seed genes are indeed significantly interconnected, forming a largest connected component of 152 out of 247 seed genes, corresponding to a *z*-score of 5.8 (empirical *p* value < 0.00001, obtained from 100k simulations of randomly selected nodes from the network). We can thus proceed to apply the random walk with restart algorithm through the dedicated UI (using restart probability *r* = 0.9, Fig. 5d). The algorithm ranks the candidate genes based on their visiting frequency. The results displayed as a bar chart in the inspection panel indicate three candidate genes as most promising (*DOK3*, *DOCK2,* and *DDX31*), all other genes having a visiting probability orders of magnitudes smaller (Fig. 5d).

Step 4: Detailed variant inspection— To further inspect the three top candidates and their relationships to the seed cluster, we first isolate all seed and candidate genes and lay them out separately. This results in a more cohesive layout, where connections between seeds and variants can be investigated more conveniently. We next inspect the top three candidate genes. The inspection panel reveals that all three genes are expressed in tissues relevant to the disease phenotype (whole blood, bone marrow, lymph node, Fig. 5e). All three also have at least one direct connection to a seed protein (DDX31: 3 seed neighbors; DOK3: 2; DOCK2: 1). We thus inspect more closely the phenotypes of the corresponding diseases caused by the immediately connected seed genes. Of the three seed genes connected to DDX31, none causes a disease with phenotypes matching those of our patient. DOK3 interacts with LCK, the respective *LCK* deficiency is characterized by several phenotypes (diarrhea, immunodeficiency, T-cell lymphopenia) closely related to those of our patient. Similarly, DOCK2 interacts with CD247, which is implicated in an immunodeficiency with similar clinical manifestations. We next inspect the functional neighborhoods of DOK3 and DOCK2 more closely, again using the dynamic transition functionality. We find that the molecular network neighborhood of DOCK2 is enriched with several processes related to immune response, such as T-cell related activities and immune synapse formation. For DOK3, on the other hand, we find no enrichment for any immune related processes.

In summary, the combined evidence of random walk rank, phenotypes associated with neighbors of known inborn errors of immunity, and functional enrichment in terms of biological process and cellular component suggests *DOCK2* as the strongest candidate. For *DDX31*, no direct neighbor shows a compatible phenotype, for *DOK3* no relationship with immune related functions could be identified. The possibility to quickly explore both local and global network neighborhoods in different functional contexts was critical for gaining these insights that could not have been obtained either purely algorithmically, nor by a conventional network analysis platform. Indeed, our platform’s unique ability to seamlessly zoom in and out between local and global connection patterns, and between different functional contexts is critical for an expert to properly evaluate the biological relevance of each piece of information.

Step 5: Post-processing—In order to follow-up on the insights gained during the VR session, we save the most relevant pieces of information for further inspection outside the VR module. The gene selection panel (Fig. 5f) can be populated with a list of arbitrary genes of interest. Through the shared data module, this list is immediately available in the web module. We can thus utilize the web module functionalities, but also any external resource for more in-depth inspections of individual genes. We further provide a clickable 2D representation of the disease neighborhood consisting of all seed and variant gene products, as well as linker proteins that connect them. A closer inspection of the top candidate DOCK2 reveals RAC1 as a neighbor. RAC1 is a key player in cytoskeletal reorganization, cell migration and adhesion. The importance of these processes for the immune response suggests a possible disease mechanism linking *DOCK2* mutations to impaired RAC1 activation and subsequent immune deficiency. This hypothesis can be experimentally tested and was indeed confirmed in the original publication of the patient^47^.

## Discussion

Biology and medicine are currently undergoing a profound transformation toward increasingly data driven fields. This transformation raises both fundamental and practical challenges on how to best utilize and interpret increasingly large and complex datasets. Our work presents a proof of concept for how Virtual Reality technology can be leveraged for this purpose. The immersive 3D environment not only drastically increases the amount of information that can be visually assessed due to the much larger available space, but also offers an interactive environment, natural to human cognition. Molecular networks are an ideal test case for exploring how VR may offer new approaches for combining genuinely human capabilities like intuition and creativity with advanced data science methodologies: First, they provide a direct and immediately interpretable visual representation (nodes and lines corresponding to gene products and their physical interactions). Second, molecular networks contain meaningful information across several scales (from individual nodes to node clusters and global connections) that can only be fully assessed when the whole network can be inspected. Third, the interpretation and relevance of specific connection patterns in the context of a concrete biological question typically requires deep, domain-specific expertise that can rarely be substituted by computational methods alone. The presented identification and functional interpretation of a genomic variant responsible for a rare disease illustrates how our platform seamlessly integrates computational tools, diverse data resources and expert interpretation of complex (patho)biological relationships.

Given the key role that molecular networks play in a wide range of biological questions, we expect that our platform will find applications beyond the specific test case presented here. Our platform can be used immediately to explore arbitrary networks and associated metadata also outside of biology. Virtual Reality, as well as Augmented and Mixed Reality technologies have recently become a major focus in the soft- and hardware industries. We hope that our work contributes to harvesting the potential of these technologies for better understanding large and complex data and building the next generation of data exploration platforms. Our modular design paradigm for separating VR interaction, data analysis, and data storage, may serve as a first step in this direction and we hope that our open-source implementation will enable a broad community to build on our work.

## Methods

### Software availability and requirements

The VRNetzer platform is freely available on https://github.com/menchelab/VRNetzer. We provide (i) an executable for the complete VRNetzer platform, (ii) a simplified desktop version that does not require dedicated VR hardware, as well as the (iii) source code for building and modifying the VRNetzer backend from scratch.

(i) The executable provides the full VRNetzer platform as a single application. The application includes the VR module for immersive visualization (requires VR hardware, see below), which is pre-configured to connect to analytics, data, and web modules hosted on a public server (requires internet access). The configuration can be modified to connect to a custom setup, for example locally hosted databases. Minimal software requirements are a MS Windows 10 operating system and the Steam VR client software, hardware requirements are detailed below.

(ii) The desktop version does not require dedicated VR hardware and instead displays the 3D content on a normal computer screen. It requires a Windows 10 operating system. The desktop version provides the first impression of our platform and can be used for exploring small to medium size networks, as well as for testing during the development of new functionalities.

(iii) The source code for the data, analytics, UI and web modules is available on github under https://github.com/menchelab/vrnetzer. The provided source code allows users to set up their own VRNetzer environment from scratch, including custom databases, to implement new data analysis methods and design own UIs.

### Hardware requirements

Our platform can be divided into a frontend (VR visualization and interaction components, see Fig. 1c) and a backend (data storage and analysis components), each with specific hardware requirements.

On the frontend side, viewing and interacting with the immersive VR environment requires a Steam VR-compatible headset with two controllers and a computer for graphics rendering. We have tested our platform on both entry level (Oculus Quest headset tethered to a standard Desktop PC), as well as high-end VR and computer hardware (HTC Vive headset on a PC with high-performance graphics card), both provide a good user experience. The primary performance requirement is the fluid display of 3D graphics, i.e., rendering high-resolution stereoscopic images at a framerate of 60 Hz or higher. Our VR implementation enables frame rates over 100 Hz for a continuous operation of all implemented network visualizations. The required computer hardware specifications of our platform are thus similar to those of other VR applications or video games with 3D graphics so that a typical computer setup for gaming will allow for a good user experience. Table 1 provides three scenarios (low cost, portable and high-end) including hardware examples and cost estimations. VR hardware has seen a steep reduction in costs over the last years. We expect this trend to continue, if not accelerate, thus making VR applications accessible to a wider audience.

**Table 1:** Three exemplary computer and VR hardware configurations for running the VRNetzer platform.

Low cost hardware setup	Portable hardware setup	High-end hardware setup
Gaming desktop PC with NVIDIA GeForce GTX 1660 SUPER graphics card (~900€)	Gaming laptop MSI GL75 10 S (~1700€)	Gaming desktop PC with NVIDIA GeForce RTX 3090 graphics card (~5000€)
VR hardware Oculus Quest (~400€)	VR hardware Oculus Quest (~400€)	VR hardware HTC Vive Pro Full Kit(~1300€)
Total cost: ~1300€	~2100€	~6300€

The left column represents a minimal stationary setup at low cost, the middle column a portable setup using a laptop computer and the right column a high-end configuration providing highest graphics quality.

The hardware requirements on the backend side depend on the particular use case. For the specific application of gene variant prioritization presented here, all analyses and database calls can be performed in around 1 s or less on any of the hardware setups described in Table 1. For the general case of using our platform to interactively visualize and analyze arbitrary network data, the performance will depend mostly on the respective datasets and analyses that are involved. To enable more demanding analyses, we designed our platform in an open and modular fashion, see below for a more detailed description of the implementation of the different components. The backend hardware can be easily adapted and scaled to the particular performance requirements of a given use case: for simple analysis tasks, the backend can run on the same computer as the frontend. For more demanding analyses, either in terms of data size or of computational power, the data and analytics modules can each be hosted on dedicated machines or even cloud-based hardware specifically optimized for a particular analysis task.

### VR module implementation

The VR module is realized using the open-source Unreal Engine 4^24^. The engine is responsible for real-time stereo rendering of the network geometry from the user’s point of view, and for fast and accurate tracking of the headset and the controllers. It also provides convenient tools for creating production-quality screenshots and videos. Our implementation makes full usage of the unique capabilities of an immersive VR environment and the respective hardware components. For example, we use the high dynamic color range and additional glow effects to guide the user’s attention to points of interest when highlighting search results or selected node clusters. We implemented a custom voxel shader for drawing large networks with hundreds of thousands of nodes and links at frame rates over 100 Hz, which are vital for the VR experience. Node positions and colors are encoded as textures so that the network rendering can be handled entirely by the GPU. To allow for interactions with the network, the voxel shader includes a C++ library for collision detection to determine where the user is pointing at. We further implemented a dynamic link fade functionality to reduce visual clutter for very dense networks: The set of all links is partitioned into random subsets that are displayed one after the other, each only for a short period of time (~1 s). This functionality is particularly convenient for exploring global network connectivities, since, at any specific time point, the overall network view remains sparse, while over time, all connections are eventually shown.

### Web module implementation

The web module is implemented in Python Flask with a JavaScript/JQuery frontend. The frontend is designed to resemble the user interaction, look, and feel of the control panel embedded in the VR module, which also allows for code sharing between the modules. Additional visualization tools, including D3.js, allow the user to interactively view small subnetworks. Importantly, the web module does not query the data module directly, but rather relies on the analytics module, described below, for data retrieval and processing. Depending on the requirements of a particular tool to be implemented, for example in terms of computational performance or data security, the analytics module can be run on the same computer as the web module, on a different local machine, or in the cloud, where it can be made globally accessible. In the event that the web and analytics modules run on the same hardware, they can be run jointly as a single web server that handles both data and HTML requests.

### Data module implementation

The data module represents the shared data storage infrastructure of the VR module and the web module. It consists of a MySQL database that communicates with the frontends via the analytics module, which is implemented as an intermediary Python Flask-based web server.

**Fig. 6:**
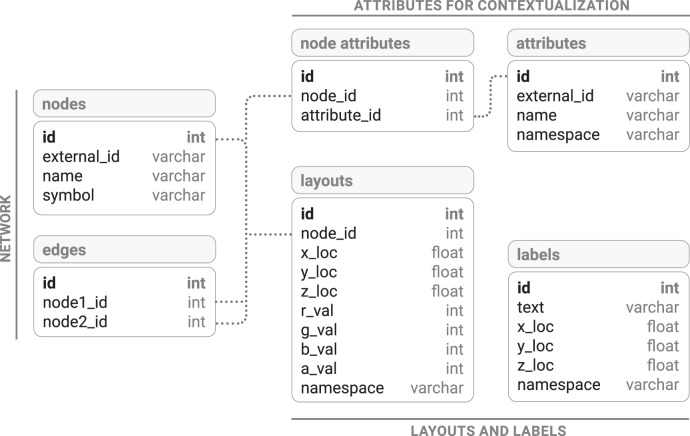
Database schema. All data are stored in a MySQL database. The user can upload their own networks using the web module to create and populate the tables automatically.

The MySQL database consists of a central schema, in which different networks are stored as separate databases, as well as one “meta” schema that stores the details of these networks. Each network schema is otherwise entirely self-contained, consisting of canonically defined tables (Fig. 6). The networks are represented by a link table and a node table. The link table is a join table with each row containing a pair of nodes. The node table contains only core, unique, and generally invariant features of a node, such as its name, symbol (if any), or external ID (if any). The rendering information for nodes, i.e., their 3D coordinates and colors in RGBA format, is stored in the layouts table. Additional information on a given node is stored by means of an attributes table (which lists every possible attribute the node can have) and a nodes-attributes table, which joins the two, and attaches an optional weight to the relationship (for instance, the level of expression of a particular gene in a specific tissue). The attribute taxonomies table stores hierarchical relationships between attributes, such as the relationships between GO terms. This allows for retrieving implicitly inherited node-attribute associations that do not have to be stored explicitly. The database can be extended or otherwise manipulated directly using standard MySQL commands. We also provide the functionality to upload new data from CSV files through a UI in the web module. The functionality performs a set of checks to ensure the consistency of the data prior to integrating it into the database.

We populated the data module with a range of widely used biological datasets: the three branches of the GO^32,33^; the disease ontology^27^ and gene-disease annotation from DisGeNET^26^ and OMIM^28^; the HPO^29–31^; pathway annotations from KeGG^11^, BioGRID^12^ and REACTOME^13^; tissue-specific gene expression data^34^; and finally PubMed article abstracts relevant to specific genes extracted from INDRA^35^.

The molecular network contained in the database has been curated from the HIPPIE database^25^, we included all interactions with an available literature reference. We included six 3D layouts: (i) a spring-based layout generated using a standard Fruchtermann–Reingold algorithm^49^; (ii) a random walk-based layout and four functional landscape layouts representing similarity according to the (iii–v) three GO branches and (vi) disease annotations, respectively (see below for details).

### Analytics module implementation

The analytics module functions as an intermediary between the VR and web-frontend modules and the database backend module. Its core function is to receive data requests from the frontend modules, perform the respective data retrieval and processing, and send the final result back to the frontend modules. The communication with the front-end modules is done via HTTP requests, and data are exchanged in the JSON format. The VR and the web module share the same data retrieval application programming interface (API), thus removing the need to implement any backend logic twice. The analytics module is written in Python, which is among the most common, accessible, and full-featured languages used for data analysis. This allows for incorporating complex analysis tasks without any need for VR programming. Our present analytics module implementation uses several common open-source packages, notably Pymysql for exchanging data with the data module, Flask for handling requests from the frontend modules, and data analysis libraries, such as SciPy^50^, Scikit-learn^51^, and NetworkX^23^. All network functionalities included in our platform are implemented using the respective NetworkX functions. For networks where node attributes are present, we also implemented gene set enrichment analysis using Fisher’s exact test and Bonferroni correction for multiple hypothesis testing.

Most commonly, the initial request to the analytics module and likewise its output consist of a set of nodes or attributes, perhaps with some numerical scoring, such as the visiting frequency for a random walk algorithm. However, our design also allows for much more general analyses. Practically all data in the database can be retrieved via the web request API, including subgraphs, enrichment analyses, articles about individual nodes, etc. As the analytics module source code is written in Python and provided with the tool, the types of analytical functionality that can be done are largely unconstrained. In general, the type of analysis done can range from very minimal (retrieval of data from the database and sending it on formatted as JSON) to substantial, such as the running of a random walk or shortest-path algorithm. Longer-running functions (on the order of seconds) are possible, as the data retrieval process is a background process in the VR and so there is no glitch in usability while the calculation is running.

### UI module implementation

The core functionality of the UI module is to send requests to the analytics module and in turn receive results and display them within the VR module. VR applications pose specific challenges regarding UI design. Conventional screen-based UIs can resort to a plethora of widely recognized visual metaphors and interaction modes via keyboard or mouse. Not all these metaphors can be translated to an immersive 3D environment, and VR-specific interaction modes are scarce and not widely recognized, in particular for applications beyond computer games. To date, only a few, often experimental data analysis applications with very specific scopes exist^52–58^. We thus designed all UI elements from scratch, combining easily recognizable 2D UI elements with intuitive, VR-specific 3D interaction modes, wherever one or the other is more appropriate to a particular task. All 2D interface panels were implemented using standard web technologies, specifically HTML and JavaScript. This enables the user to customize our platform and create new interface panels without specialized knowledge in VR game engine programming. A wide variety of web-based technologies are thus readily available, such as state-of-the-art dashboard interaction panels, using for example D3.js. This design further facilitates the integration of VR module interfaces into the web module and increases the performance by unloading the game thread/VR module.

Note that communication between the VR module and the UI module can also be used to create a detailed record of a user’s VR exploration session. This wealth of data could yield interesting insights into the process of visual network analytics and help design more efficient visualization and interaction tools. We have implemented a prototype for such a comprehensive tracing functionality which is described in the online documentation.

### Functional network landscapes

We introduce the following procedure for generating 3D network layouts, in which node coordinates are based on their functional annotations: We start by constructing an *n* × *m* feature matrix ***F***, where *n* is the number of nodes in the network, and *m* the number of different features that may be assigned to a specific node, for example, the total number of GO terms. The matrix is populated with *F*_*ij*_ = 1, if node *i* is annotated with feature *j* (i.e., gene *i* is associated with GO term *j*) and *F*_*ij*_ = 0, if not. We then use the dimensionality reduction method tSNE^59^ to embed the nodes into 3D space, such that the distance between nodes reflects the cosine similarity of their respective feature vectors. This results in a functional landscape, where clusters of nodes emerge that correspond to groups of genes with similar functional annotations. Finally, we perform a gene set enrichment analysis (using Fisher’s exact test and Bonferroni correction for multiple hypothesis testing) to determine the particular annotations that are also displayed next to the cluster.

We used the procedure to generate layouts and corresponding annotations based on the three GO branches (biological process, molecular function, and cellular component), as well as a disease layout based on disease annotations.

We used a slightly modified procedure to generate a 3D layout that is purely based on network structure: Here, we populated the matrix ***F****,* such that *F*_*ij*_ is given by the frequency with which node *j* is visited by a random walker starting from node *i* with restart probability *r* = 0.9.

Finally, we also included a spring-based layout from a Fruchtermann–Reingold algorithm implementation^49^.

### Comparison with general network analysis tools

There is a plethora of network analysis softwares available that offer a wide range of functionalities. Table 2 shows a selection emphasizing the rich diversity among existing tools in terms of the following specifications (represented in the columns from left to right): (i) Software platform, e.g., standalone or web-based software, availability of graphical or programmatic UIs. (ii) Network visualization type, e.g., static or interactive, 2D or 3D (‘projected 3D’ refers to 3D renderings on a 2D screen). (iii) Fluid interactions with small, medium, and genome-scale networks, as tested on the full interactome used in this study and on random samples of 150 (small) and 1500 (medium) nodes, respectively. As a minimal criterion for fluid interaction, we required that the user can smoothly zoom in and out of the network using the same hardware setup as our VR platform. (iv) Possibility to add custom code for data and network analysis, e.g., via a provided API or direct code injection. (v) Possibility to modify or add custom UIs, e.g., via plugins or open UI source code. (vi) Options for incorporating external datasets, e.g., by connecting a database or importing text files. (vii) Availability of source code.

**Table 2:** Comparison of selected network analysis and visualization tools.

Tool	Description and platform	Network visualization	Network exploration	Custom network analyses	Custom User Interface design	External data	Open source
VRNetzer	VR platform for visualizing and exploring large-scale networks	True 3D;VR	Interactive;Genome-scale networks	Via API^a^	Open, web-based UI design	File import or database connection	Yes
Cytoscape^17^	Standalone software for network visualization and analysis	2D; projected 3D via plugins	Interactive;Genome-scale networks^b^	Via API or plugins	Limited UI design of plugins	File import or database connection	Yes
Gephi^18^	Standalone software for network visualization and analysis	2D	Interactive;medium-scale networks	Via API or plugins	Limited UI design of plugins	File import	Yes
Graphia^19^	Standalone software for network visualization and analysis	2D; projected 3D	Interactive;Genome-scale networks	Via plugins	No	File import	Yes
OmicsNet^20^	Web-based software for visualization and analysis of molecular networks	2D; projected 3D	Interactive;medium-scale networks	No	No	File import	No
GraphVis^21^	Web-based software for network visualization and analysis	2D	Interactive;medium-scale networks	No	No	Online network repository	No
STRING^14^	Web-based resource for analysis and visualization of protein interaction networks	2D	Interactive;small-scale networks^b^	No	No	No	No
Graphviz^22^	Command-line based tools for graph layout and drawing.	2D	Static images	Via API	VIA API	File import or via API	Yes
NetworkX^23^	Python package for network analysis; includes layout drawing functionalities	2D; projected 3D	Static images	Via python code	Open UI design via auxiliary packages	File import or database connection	Yes

^a^*API* application programming interface.

^b^small (medium) = random samples of 150 (1500) nodes from the interactome network used in this study.

The single most important and distinguishing feature of our platform is the VR network visualization and resulting capability to interactively explore large-scale networks. In addition, the VRNetzer platform is uniquely designed to allow for modifying each aspect of software, ranging from the analysis methods to the UI, as well as the underlying hardware, ranging from a single workstation to distributed cloud servers.

### Comparison with gene annotation and prioritization tools

Several of the general tools introduced in Table 2 can in principle be customized to perform network-based variant prioritization. However, depending on the scope of the respective software, this may require substantial adaptations. There are also specialized solutions available that may be more convenient for the particular purpose of gene annotation and prioritization. In the following, we discuss three of such specialized tools that are widely used and can exemplarily highlight key features that distinguish our implementation:

*ToppGene*^44^ is a web portal that offers a variety of functionalities, including gene prioritization. Candidate genes can be prioritized based on functional annotations or using purely network-based strategies, including an adapted version of the PageRank algorithm which closely resembles the random walk method used in this study. These methods have been demonstrated to be effective for common, polygenic diseases, and should in principle also be applicable in the context of rare, monogenic diseases. Also functional enrichment can be performed using diverse annotation data, many of which are also included in our platform (e.g., GO, human and mouse phenotypes, pathway data, gene expression). There are three major differences to our platform: First, the pipeline of ToppGene does not include any interactive network analyses. Second, there is no functionality for uploading own datasets. The protein–protein interaction network used by ToppGene is based on data from BIND (Biomolecular Interaction Network Database) with a filter that results in a high-confidence, yet relatively small network of only 8340 genes and 27,250 interactions. Third, no alternative prioritization methods or additional analyses can be implemented.

Similar restrictions apply to the *The Human Gene Connectome* (HGC)^45^, a dedicated rare disease gene variant prioritization tool which uses the same basic methodology as implemented in our exemplary application: A given set of candidate genes (e.g., variant genes) is ranked by their respective network-based proximity to a set of core genes (e.g., seed genes known to be associated with a particular phenotype). HGC relies on the STRING (Search Tool for the Retrieval of Interacting Genes/Proteins) network and uses the shortest path length to define the biological distance between core genes and candidates. No customized network data can be uploaded, and no alternative prioritization algorithms can be used. The pipeline does not include any interactive network visualizations or analyses and provides output in the form of a simple table.

A popular web resource that includes a convenient and interactive 2D network visualization is given by *STRING* ^14^. STRING is a comprehensive resource for known and predicted protein–protein interactions and allows for visualizing and exploring networks of selected proteins. While STRING does not offer a dedicated variant prioritization pipeline, the included functionalities, such as clustering nodes within subnetworks and performing enrichment analysis using various built-in resources (e.g., GO, Pfam, and KEGG) can also be applied in the context of network-based prioritization. However, as any 2D network visualization tool, these analyses are critically limited by the size of the considered subnetworks, larger networks cannot be meaningfully visualized and explored.

Variant prioritization often involves larger networks, since the respective network neighborhoods of all candidate genes need to be evaluated. Due to the small-world property of the interactome even relatively small seed and variant gene sets may lead to a very large number of connected neighbors, depending on the exact neighborhood identification strategy: Fig. 4a shows the interactions between seed and variant genes in the interactome. Containing 157 out of 260 genes, the largest connected component is significantly larger than expected by chance for the same number of randomly selected genes (*z*-score = 6.1, empirical *p* value < 0.00001; obtained from 100k simulations). This indicates that the genes reside in a particular interactome neighborhood and serves as a basic test for the applicability of approaches that aim to explore this neighborhood. Figure 4b–d illustrates different strategies for constructing such extended neighborhoods: A minimal neighborhood can be constructed using a Steiner tree algorithm^60^ which identifies a minimal set of linker genes that connect all initial genes into a single component (Fig. 4b). The resulting neighborhood is compact enough to be further investigated using conventional tools. However, the compact size with only a few additional genes also limits the interpretation, in particular regarding the biological processes involving the variant genes at the periphery. Alternative approaches that result in broader, more informative neighborhoods are to consider all first neighbors (Fig. 4c) or to prioritize nodes using the random walk-based method described in the manuscript (Fig. 4d). In both cases, the size and complexity of the resulting subnetworks lead to 2D visualizations that can hardly be interpreted and offer little insights. The immersive 3D perspective of our platform, on the other hand, allows for interactive exploration not only of such subnetworks but of the entire interactome.

### Reporting summary

Further information on research design is available in the Nature Research Reporting Summary linked to this article.

## Supplementary information

Reporting Summary

## Data Availability

All data used in this study are publicly available and were downloaded from the websites of the referenced original studies. Weblinks, version numbers and release dates can be found in the data availability section on https://github.com/menchelab/VRNetzer.

## Code availability

Source code and pre-compiled executables for the VRNetzer platform are available on https://github.com/menchelab/VRNetzer^61^. We used open-source softwares and programming environments Python (version 3.6), JavaScript (version ES6), MySQL (version 8.0), as well as the EPIC Unreal engine (version 4). Detailed software and package dependencies can be found in the dependencies section on https://github.com/menchelab/VRNetzer.

## References

[1] Menche J (2015). Disease networks. Uncovering disease-disease relationships through the incomplete interactome. Science.

[2] Bergthaler A, Menche J (2017). The immune system as a social network. Nat. Immunol..

[3] Caldera M, Buphamalai P, Müller F, Menche J (2017). Interactome-based approaches to human disease. Curr. Opin. Syst. Biol..

[4] McGillivray P (2018). Network analysis as a grand unifier in biomedical data science. Annu. Rev. Biomed. Data Sci..

[5] Costanzo M (2016). A global genetic interaction network maps a wiring diagram of cellular function. Science.

[6] Piñero J, Berenstein A, Gonzalez-Perez A, Chernomoretz A, Furlong LI (2016). Uncovering disease mechanisms through network biology in the era of next generation sequencing. Sci. Rep..

[7] Huttlin EL (2017). Architecture of the human interactome defines protein communities and disease networks. Nature.

[8] Luck K (2020). A reference map of the human binary protein interactome. Nature.

[9] Ghiassian SD, Menche J, Barabási A-L (2015). A DIseAse MOdule Detection (DIAMOnD) algorithm derived from a systematic analysis of connectivity patterns of disease proteins in the human interactome. PLoS Comput. Biol..

[10] Orchard S (2014). The MIntAct project—IntAct as a common curation platform for 11 molecular interaction databases. Nucleic Acids Res..

[11] Kanehisa M, Sato Y, Furumichi M, Morishima K, Tanabe M (2019). New approach for understanding genome variations in KEGG. Nucleic Acids Res..

[12] Oughtred R (2019). The BioGRID interaction database: 2019 update. Nucleic Acids Res..

[13] Jassal B (2020). The reactome pathway knowledgebase. Nucleic Acids Res..

[14] Szklarczyk, D. et al. The STRING database in 2021: customizable protein-protein networks, and functional characterization of user-uploaded gene/measurement sets. *Nucleic Acids Res.*10.1093/nar/gkaa1074 (2020). 10.1093/nar/gkaa1074 PMC7779004 33237311

[15] Köberlin MS (2015). A conserved circular network of coregulated lipids modulates innate immune responses. Cell.

[16] Caldera M (2019). Mapping the perturbome network of cellular perturbations. Nat. Commun..

[17] Shannon P (2003). Cytoscape: a software environment for integrated models of biomolecular interaction networks. Genome Res..

[18] Bastian, M., Heymann, S. & Jacomy, M. Gephi: an open source software for exploring and manipulating networks. in *Third international AAAI Conference on Weblogs and Social Media* (aaai.org, 2009).

[19] Freeman, T. C. et al. *Graphia: a Platform for the Graph-based Visualisation and Analysis of Complex Data*. (Cold Spring Harbor Laboratory, 2020). 10.1101/2020.09.02.279349.

[20] Zhou G, Xia J (2018). OmicsNet: a web-based tool for creation and visual analysis of biological networks in 3D space. Nucleic Acids Res..

[21] Ahmed, N. & Rossi, R. Interactive visual graph analytics on the web. ICWSM **9** (AAAI Press, Palo Alto, California USA, 2015).

[22] Ellson, J., Gansner, E., Koutsofios, L., North, S. C. & Woodhull, G. Graphviz—open source graph drawing tools. in *International Symposium on Graph Drawing,* 483–484 (Springer, 2001).

[23] Hagberg, A., Swart, P. & S Chult, D. Exploring network structure, dynamics, and function using networkx. https://www.osti.gov/biblio/960616 (2008).

[24] EPIC GAMES. Unreal engine 4. https://www.unrealengine.com/ (2015).

[25] Alanis-Lobato G, Andrade-Navarro MA, Schaefer MH (2017). HIPPIE v2.0: enhancing meaningfulness and reliability of protein-protein interaction networks. Nucleic Acids Res..

[26] Piñero J (2017). DisGeNET: a comprehensive platform integrating information on human disease-associated genes and variants. Nucleic Acids Res..

[27] Schriml LM (2019). Human disease ontology 2018 update: classification, content and workflow expansion. Nucleic Acids Res..

[28] Amberger JS, Bocchini CA, Schiettecatte F, Scott AF, Hamosh A (2015). OMIM.org: Online Mendelian Inheritance in Man (OMIM®), an online catalog of human genes and genetic disorders. Nucleic Acids Res..

[29] Robinson PN (2008). The human phenotype ontology: a tool for annotating and analyzing human hereditary disease. Am. J. Hum. Genet..

[30] Köhler S (2017). The human phenotype ontology in 2017. Nucleic Acids Res..

[31] Köhler S (2019). Expansion of the human phenotype ontology (HPO) knowledge base and resources. Nucleic Acids Res..

[32] Ashburner, M. et al. Gene ontology: tool for the unification of biology. *Nat. Genet.* **25**, 25–29 (2000). 10.1038/75556 PMC3037419 10802651

[33] The Gene Ontology Consortium. (2019). The gene ontology resource: 20 years and still GOing strong. Nucleic Acids Res..

[34] Uhlén M (2015). Proteomics. Tissue-based map of the human proteome. Science.

[35] Gyori BM (2017). From word models to executable models of signaling networks using automated assembly. Mol. Syst. Biol..

[36] Lovász, L. et al. Random walks on graphs: a survey. In (eds Miklós, D., Sós, V. T. & Szőnyi, T.) *Combinatorics, Paul Erdös is Eighty* Vol. 2, 1–46 (János Bolyai Mathematical Society, Budapest, Hungary, 1993).

[37] Blomen VA (2015). Gene essentiality and synthetic lethality in haploid human cells. Science.

[38] Bartha I, di Iulio J, Venter JC, Telenti A (2018). Human gene essentiality. Nat. Rev. Genet..

[39] Hu, J. X., Thomas, C. E. & Brunak, S. Network biology concepts in complex disease comorbidities. *Nat. Rev. Genet.*10.1038/nrg.2016.87 (2016). 10.1038/nrg.2016.87 27498692

[40] Köhler S, Bauer S, Horn D, Robinson PN (2008). Walking the interactome for prioritization of candidate disease genes. Am. J. Hum. Genet..

[41] Smedley D (2014). Walking the interactome for candidate prioritization in exome sequencing studies of Mendelian diseases. Bioinformatics.

[42] Smedley D (2015). Next-generation diagnostics and disease-gene discovery with the Exomiser. Nat. Protoc..

[43] Robinson S (2017). Incorporating interaction networks into the determination of functionally related hit genes in genomic experiments with Markov random fields. Bioinformatics.

[44] Chen J, Bardes EE, Aronow BJ, Jegga AG (2009). ToppGene suite for gene list enrichment analysis and candidate gene prioritization. Nucleic Acids Res..

[45] Itan Y (2014). HGCS: an online tool for prioritizing disease-causing gene variants by biological distance. BMC Genom..

[46] Feldman I, Rzhetsky A, Vitkup D (2008). Network properties of genes harboring inherited disease mutations. Proc. Natl Acad. Sci. USA.

[47] Dobbs K (2015). Inherited DOCK2 deficiency in patients with early-onset invasive infections. N. Engl. J. Med..

[48] Bousfiha A (2018). The 2017 IUIS phenotypic classification for primary immunodeficiencies. J. Clin. Immunol..

[49] Fruchterman TMJ, Reingold EM (1991). Graph drawing by force-directed placement. Softw. Pract. Exp..

[50] Virtanen P (2020). SciPy 1.0: fundamental algorithms for scientific computing in Python. Nat. Methods.

[51] Pedregosa F (2011). Scikit-learn: machine learning in python. J. Mach. Learn. Res..

[52] Gillet A, Sanner M, Stoffler D, Olson A (2005). Tangible interfaces for structural molecular biology. Structure.

[53] Haug-Baltzell A, Stephens SA, Davey S, Scheidegger CE, Lyons E (2017). SynMap2 and SynMap3D: web-based whole-genome synteny browsers. Bioinformatics.

[54] Goddard TD (2018). Molecular visualization on the Holodeck. J. Mol. Biol..

[55] O’Connor M (2018). Sampling molecular conformations and dynamics in a multiuser virtual reality framework. Sci. Adv..

[56] Stefani C, Lacy-Hulbert A, Skillman T (2018). ConfocalVR: immersive visualization for confocal microscopy. J. Mol. Biol..

[57] Legetth, O. et al. CellexalVR: A virtual reality platform for the visualisation and analysis of single-cell gene expression data. preprint at https://www.biorxiv.org/content/10.1101/329102v3 (2019).

[58] Xu, K. et al. VRmol: an integrative cloud-based virtual reality system to explore macromolecular structure. *Bioinformatics*, btaa696, 10.1093/bioinformatics/btaa696 (2020). 10.1093/bioinformatics/btaa696 32745209

[59] Maaten Lvander, Hinton G (2008). Visualizing data using t-SNE. J. Mach. Learn. Res..

[60] Zheng S, Zhao Z (2012). GenRev: exploring functional relevance of genes in molecular networks. Genomics.

[61] Pirch, S. et al., The VRNetzer platform enables interactive network analysis in virtual reality, Zenodo, 10.5281/zenodo.4597735 (2021). 10.1038/s41467-021-22570-w PMC8065164 33893283

