# Supplementary material for: The VRNetzer platform enables interactive network analysis in Virtual Reality
Source: Nat Commun. 2021 Apr 23;12:2432. doi: 10.1038/s41467-021-22570-w (PMC8065164; doi:10.1038/s41467-021-22570-w)
Supplement: Supplementary file 1 — Reporting Summary [file 41467_2021_22570_MOESM1_ESM.pdf]

nature research

Corresponding author(s): Jörg Menche
Last updated by author(s): Mar 4, 2021

Reporting Summary

Nature Research wishes to improve the reproducibility of the work that we publish. This form provides structure for consistency and transparency in reporting. For further information on Nature Research policies, see our Editorial Policies and the Editorial Policy Checklist.

Statistics

For all statistical analyses, confirm that the following items are present in the figure legend, table legend, main text, or Methods section.

n/a	Confirmed	
☒	☐	The exact sample size (*n*) for each experimental group/condition, given as a discrete number and unit of measurement
☒	☐	A statement on whether measurements were taken from distinct samples or whether the same sample was measured repeatedly
☒	☐	The statistical test(s) used AND whether they are one- or two-sided<br>*Only common tests should be described solely by name; describe more complex techniques in the Methods section.*
☒	☐	A description of all covariates tested
☒	☐	A description of any assumptions or corrections, such as tests of normality and adjustment for multiple comparisons
☒	☐	A full description of the statistical parameters including central tendency (e.g. means) or other basic estimates (e.g. regression coefficient) AND variation (e.g. standard deviation) or associated estimates of uncertainty (e.g. confidence intervals)
☒	☐	For null hypothesis testing, the test statistic (e.g. *F*, *t*, *r*) with confidence intervals, effect sizes, degrees of freedom and *P* value noted<br>*Give P values as exact values whenever suitable.*
☒	☐	For Bayesian analysis, information on the choice of priors and Markov chain Monte Carlo settings
☒	☐	For hierarchical and complex designs, identification of the appropriate level for tests and full reporting of outcomes
☒	☐	Estimates of effect sizes (e.g. Cohen's *d*, Pearson's *r*), indicating how they were calculated

Our web collection on statistics for biologists contains articles on many of the points above.

Software and code

Policy information about availability of computer code

Data collection	All data used in this study are publicly available and were downloaded from the websites of the original studies / resources, see Data section below for a detailed list.
Data analysis	Source code and pre-compiled executables for the VRNetzer platform are available on https://github.com/menchelab/VRNetzer. We used open source softwares and programming environments Python (version 3.6), JavaScript (version ES6), MySQL (version 8.0), as well as the EPIC Unreal engine (version 4). Detailed software and package dependencies can be found in the dependencies section on https://github.com/menchelab/VRNetzer.

For manuscripts utilizing custom algorithms or software that are central to the research but not yet described in published literature, software must be made available to editors and reviewers. We strongly encourage code deposition in a community repository (e.g. GitHub). See the Nature Research guidelines for submitting code & software for further information.

Data

Policy information about availability of data

All manuscripts must include a data availability statement. This statement should provide the following information, where applicable:
- Accession codes, unique identifiers, or web links for publicly available datasets
- A list of figures that have associated raw data
- A description of any restrictions on data availability

All data used in this study are publicly available and were downloaded from the websites of the referenced original studies.
We used the following publicly available data sources:
Gene ontology (GO) taxonomy, from http://geneontology.org/docs/download-ontology/
GO annotations, from http://current.geneontology.org/products/pages/downloads.html

Disease ontology (DO) taxonomy, from https://github.com/DiseaseOntology/HumanDiseaseOntology/tree/main/src/ontology/releases
DisGeNET, from https://www.disgenet.org/downloads
Online Mendelian Inheritance in Man (OMIM), from https://www.omim.org/
Human Phenotype Ontology (HPO) annotation, from https://hpo.jax.org/app/download/annotation
HPO taxonomy, from https://hpo.jax.org/app/download/ontology
Kyoto Encyclopedia of Genes and Genomes (KeGG), from https://www.genome.jp/kegg/genes.html
Biological General Repository for Interaction Datasets (BioGRID), from https://downloads.thebiogrid.org/
REACTOME, from https://reactome.org/download-data
Genotype-Tissue Expression (GTEx) data, from https://gtexportal.org/home/datasets
Pubmed articles from INDRA, from https://github.com/sorgerlab/indra
Human Integrated Protein-Protein Interaction Reference (HIPPIE) PPIs, from http://cbdm-01.zdv.uni-mainz.de/~mschaefer/hippie/download.php

Weblinks, version numbers and release dates can also be found in the data availability section on https://github.com/menchelab/VRNetzer.

Field-specific reporting

Please select the one below that is the best fit for your research. If you are not sure, read the appropriate sections before making your selection.

[x] Life sciences [ ] Behavioural & social sciences [ ] Ecological, evolutionary & environmental sciences

For a reference copy of the document with all sections, see nature.com/documents/nr-reporting-summary-flat.pdf

Life sciences study design

All studies must disclose on these points even when the disclosure is negative.

Sample size	Study does not involve any analyses to which sample size calculations are applicable.
Data exclusions	No data were excluded in the study.
Replication	Study does not involve experimental results.
Randomization	Study does not involve any samples / organisms / participants.
Blinding	Study does not involve any samples / organisms / participants.

Reporting for specific materials, systems and methods

We require information from authors about some types of materials, experimental systems and methods used in many studies. Here, indicate whether each material, system or method listed is relevant to your study. If you are not sure if a list item applies to your research, read the appropriate section before selecting a response.

Materials & experimental systems

n/a	Involved in the study	
[x]	[ ]	Antibodies
[x]	[ ]	Eukaryotic cell lines
[x]	[ ]	Palaeontology and archaeology
[x]	[ ]	Animals and other organisms
[x]	[ ]	Human research participants
[x]	[ ]	Clinical data
[x]	[ ]	Dual use research of concern

Methods

n/a	Involved in the study	
[x]	[ ]	ChIP-seq
[x]	[ ]	Flow cytometry
[x]	[ ]	MRI-based neuroimaging
